# Multilevel comparative analysis of the contributions of genome reduction and heat shock to the *Escherichia coli* transcriptome

**DOI:** 10.1186/1471-2164-14-25

**Published:** 2013-01-16

**Authors:** Bei-Wen Ying, Shigeto Seno, Fuyuro Kaneko, Hideo Matsuda, Tetsuya Yomo

**Affiliations:** 1 Graduate School of Information Science and Technology, Osaka University, 1-5 Yamadaoka, Suita, Osaka, 565-0871, Japan; 2 Graduate School of Frontier Biosciences, Osaka University, 1-5 Yamadaoka, Suita, Osaka, 565-0871, Japan; 3 Exploratory Research for Advanced Technology (ERATO), Japan Science and Technology Agency (JST), Suita, Osaka, 565-0871, Japan

**Keywords:** Transcriptome, Negative epistasis, Genome reduction, Chromosomal periodicity, Regulatory network, Transcriptional change, Genomic interruption, Environmental perturbation, Heat shock, Directionality

## Abstract

**Background:**

Both large deletions in genome and heat shock stress would lead to alterations in the gene expression profile; however, whether there is any potential linkage between these disturbances to the transcriptome have not been discovered. Here, the relationship between the genomic and environmental contributions to the transcriptome was analyzed by comparing the transcriptomes of the bacterium *Escherichia coli* (strain MG1655 and its extensive genomic deletion derivative, MDS42) grown in regular and transient heat shock conditions.

**Results:**

The transcriptome analysis showed the following: (i) there was a reorganization of the transcriptome in accordance with preferred chromosomal periodicity upon genomic or heat shock perturbation; (ii) there was a considerable overlap between the perturbed regulatory networks and the categories enriched for differentially expressed genes (DEGs) following genome reduction and heat shock; (iii) the genes sensitive to genome reduction tended to be located close to genomic scars, and some were also highly responsive to heat shock; and (iv) the genomic and environmental contributions to the transcriptome displayed not only a positive correlation but also a negatively compensated relationship (*i*.*e*., antagonistic epistasis).

**Conclusion:**

The contributions of genome reduction and heat shock to the *Escherichia coli* transcriptome were evaluated at multiple levels. The observations of overlapping perturbed networks, directional similarity in transcriptional changes, positive correlation and epistatic nature linked the two contributions and suggest somehow a crosstalk guiding transcriptional reorganization in response to both genetic and environmental disturbances in bacterium *E*. *coli*.

## Background

The bacterial transcriptome, as one of the important genome-level information, reflects the global cellular activity (*i*.*e*., fitness). Since the fitness and/or activity of the cells is directly influenced by the environmental perturbation (*e*.*g*., heat or cold stress) and genetic disturbance (*e*.*g*., mutations or deletions in genome), the evaluation of the environmental and genetic contributions to transcriptome turns to be highly essential. In recent, the transcriptome analysis (*i*.*e*., gene expression profiling) of bacteria (*e*.*g*., *Escherichia coli*) has been performed extensively using a range of experimental and analytical tools [1-7], to illustrate an overall picture of the biochemical events occurring inside the cells. In particular, to examine the environmental contributions to bacterial transcriptome, gene expression profiling has been tested across multiple environmental conditions in a high-throughput manner [8-10]. As a consequence, the intensive studies on global transcriptional activity contributed by environmental perturbations successfully provided conclusions regarding the molecular mechanisms involved in specific pathways and/or regulatory networks [9,11-14], as well as the theoretical and/or overall outlook in regard to global dynamic changes in transcriptome [15].

However, the genetic contribution to bacterial transcriptome has been rarely reported. Although the previous studies revealed the potential links between transcriptional regulation and chromosome organization [16] and the relation between changes in DNA methylation and chromosomal structure and changes in global gene expression [3,5], whether and how the genomic scars (*e*.*g*., deletions) influence the global transcriptional activity is unclear.

Both genomic and environmental perturbations could initiate a global change in the transcriptome, because both are stressful for living cells. Whether there is any potential linkage between the two contributions is an intriguing question. To address the question, a comprehensive analysis comparing the effects of genomic and environmental interruptions on the transcriptome is highly required. Such analysis would not only enable a comparison of the internal and external influences on the plasticity of genome-wide transcriptional activity but would also provide a valuable example of a global and conceptual assessment of high-throughput biological data. Therefore, a multi-level survey of genomic expression is highly in demand, to show how genomic disturbance causes the global transcriptional reorganization and whether there is any relationship between the contributions of genomic and environmental interruptions to transcriptome.

As a pilot study, we evaluated these two distinct entities, the genome and the environment, at the level of the transcriptome. We addressed how genomic and environmental perturbations contribute to the bacterial transcriptome and whether there is any interaction between the two influences. A multiple deletion *E*. *coli* strain, MDS42 [17], was compared with its wild-type parent strain MG1655 [18,19] in this study. Because of its lack of insertion sequences (ISs) [17,20], MDS42 has been widely used in a number of applications [21-25]; however, its detailed, genome-wide analysis has been rarely reported [21-25]. The genes used for the comparative transcriptome analysis were selected on the basis of the genome sequences of MDS42 and MG1655. The comparison of MG1655 to MDS42 provided insights into genomic disturbance-induced transcriptional reorganization. The evaluation of the heat shock response (a transient response to an elevated temperature) provided insights into environmental perturbation-induced transcriptional changes. We examined whether the losses of insertion elements in genome interrupt the genome-wide transcriptional activity (*i*.*e*., the transcriptome) and whether these multiple deletions in genome and the heat shock stress exert common or different effects on transcriptional changes. Furthermore, we performed multilevel comparative analyses of chromosomal periodicity, regulatory networks, the localization of differential gene expression, transcriptional sensitivity in response to genomic and environmental perturbations and the epistatic effect, that is, a negatively compensated relationship, on the transcriptome of the genome and the environment.

## Results

### Strains and genes subjected to transcriptome analysis

**Figure 1:**
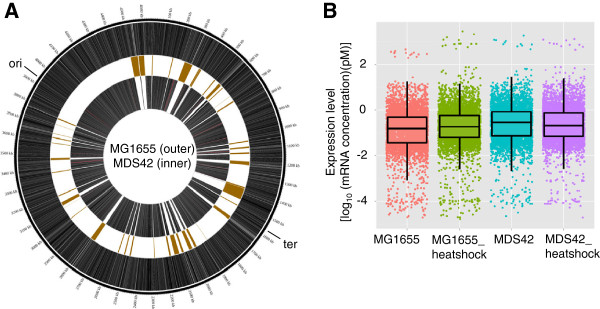
**Overview of genomes and transcriptomes. A**. Circle diagram of the MG1655 and MDS42 genomes. The genes present in MG1655 and MDS42 are shown in the outer and inner rings, respectively, and visualized using Circos [30]. The gold boxes indicate the deleted segments that were originally reported [17]. The point mutations in MG1655 are indicated with red tick marks. The origin and terminus of replication are indicated outside of the circle. **B**. Box plot of gene expression in MG1655 and MDS42. The average expression levels of 4428 and 3710 genes in MG1655 and MDS42, in exponential phase growth or under heat shock conditions, are shown in pink (MG1655), green (MG1655_heatshock), cyan (MDS42) and purple (MDS42_heatshock), respectively. The expression levels are represented by the log-scale mRNA concentrations. The dots represent the genes.

Gene expression was analyzed using a custom, high-density DNA microarray (a single nucleotide tiling array) with a precise and wide quantitative range of mRNA concentrations [26,27], as previously described for genome resequencing [28] and transcriptome analysis [29]. First, the wild-type strain MG1655 and the multiple deletion strain MDS42 [17] were used to examine whether and how removing the nonessential genomic regions in *E*. *coli* contributed to its transcriptome. Repeated experiments were performed with cells grown precisely to mid-exponential phase in minimal medium. The genes shared between the two strains were determined by comparing their genome sequences (MDS42: DDBJ No. AP012306; MG1655: GenBank No. U00096). In total, locus tags (b numbers) were assigned to 4485 and 3778 open reading frames (ORFs) in MG1655 and MDS42, respectively (Figure 1A), which was consistent to the original report [17]. Genes with repeated locus tags were removed from the analysis, for a final total of 4428 genes with locus tags in MG1655. Excluding the 696 deleted genes [17] and 22 mutated genes in MG1655 (determined by comparing the genome sequences, see the Materials and Methods), a total of 3710 genes were determined to be common between the two strains. Subsequently, as the heat shock stress is known as one of many external perturbations and showed distinguishable expression profiles [9,11], heat shock experiments were performed to evaluate how the external disturbance contributed to the transcriptome for comparative analyses. The average gene expression levels from repeated experiments were plotted in Figure 1B and used for further analysis. We showed that neither genomic reduction nor heat shock altered the shape of the distribution of gene expression (Figure 1B).

### Priority in chromosomal periodicity

**Figure 2:**
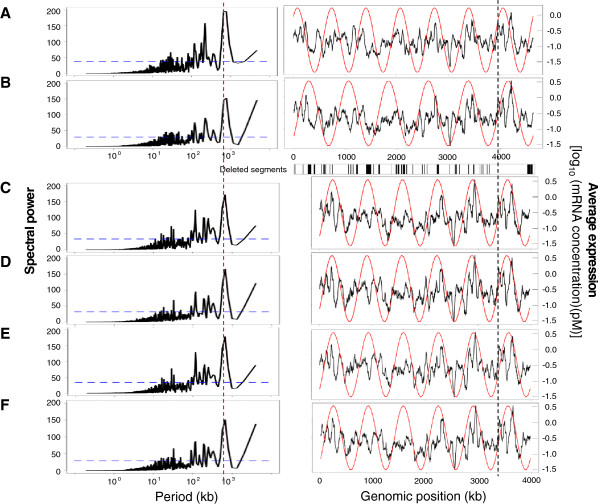
**Periodicity of transcriptional activity.** The periodograms of the average transcriptional levels of MG1655 at 37°C (**A**), MG1655 following heat shock (**B**), MDS42 at 37°C (**C**), and MDS42 following heat shock (**D**), with the highest (major) peaks at 663, 773, 663, and 663 kb, respectively. The vertical, broken line in red indicates the major peak at 663 kb. The significant periodicities at the 95% confidence level are indicated by blue dashed lines. The expression levels of 4428 and 3710 genes were plotted for MG1655 and MDS42, respectively. In addition, the periodicities of the expression of the 3710 common genes in MG1655 at 37°C (**E**) and following heat shock (**F**) are plotted against the MDS42 genome. The average transcriptional levels of the genes for every 100-bp sliding window are shown as black lines, and the corresponding periodicities are shown as red curves. The location of the origin of replication (*ori*) is indicated by the black broken line, and the deleted segments from MG1655 are indicated by black bars. The average expression represents the log-scale mRNA concentration. The spectral power calculated with Fourier transform indicates the strength of the periodicity of the average expression along the genome.

The periodicity of genome-wide transcriptional activity was studied to provide a global view of gene expression profiling [4,6,31,32]. The average transcription levels (*i*.*e*., mRNA concentrations) over the entire genome were plotted for both genomes using a 100-bp sliding window (Figure 2). The periodic patterns showed a major peak of approximately 663 kb (Figure 2A) in MG1655, consistent with previous reports [4,32]. However, these periodic patterns were disturbed by heat shock. The most significant wavelength, as indicated by the red, vertical, broken line in Figure 2 (left panels), shifted from 663 to 773 kb (to the right of the red line, a close-up view can be find in Additional file 1: Figure S4). This slight but statistically significant change in the major peak caused a reduction in the number of periods from seven to six (Figure 2A–B, right panels). This finding indicated that the heat shock treatment initiated a reorganization of the transcriptome that gave a high priority to a periodicity of six periods.

Interestingly, the reduced genome also showed a fixed periodicity of six periods. MDS42 maintained the parental periodogram, with a major peak at 663 kb that was accompanied by a decrease in the chromosomal period number (Figure 2C). This reduced period was unchanged in MDS42 in response to heat shock perturbation (Figure 2D). In addition, when the expression of the 3710 common genes in MG1655 was plotted against the MDS42 genome, an identical chromosomal periodicity of six periods was observed independently of external conditions (Figure 2E and F). Statistical analyses confirmed that the altered periodicity was highly specific for the MDS42 genome and was not due to random deletions in the genome (*P* < 0.001). The stable six periods may be have been caused by the presence of six macrodomains in the chromosomal structure, as determined by recombination activity [33]. The results suggested that the absent genetic regions in MDS42 (Figure 1A, gold boxes) may contain a considerable number of genes that are not or rarely transcribed under heat shock conditions, potentially contributing to an additional period. Thus, the multiple deletions not only reduced the genome size but also provided, probably by chance, a steady periodicity to the global transcriptional activity of the *E*. *coli* chromosome.

### Overlaps in perturbed regulatory networks

**Figure 3:**
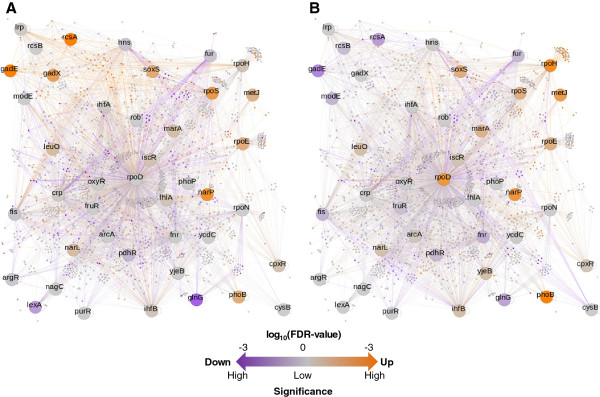
**Transcriptional changes in regulatory networks.** The information regarding the regulatory networks is from RegulonDB. The genes under the control of the same transcription factor (TF) or sigma factor were assigned to the same group. The nodes and arcs represent genes and regulation, respectively. The large nodes denote sigma factors or regulators that control more than 15 genes. The genes with conserved expression are shown in gray, and those with notable transcriptional changes are shown in color. The increased and decreased expression levels resulting from genome reduction (**A**) or heat shock (**B**) are quantitatively marked as gradations, as indicated in the scale bar. Vivid orange indicate highly significant increases in expression, and dark purple represent highly significant decreases in expression.

Regulatory network maps comprising the transcription factors (TFs, or regulators) that control more than 15 genes (42 of 175 regulatory networks) were constructed as shown in Figure 3. Significant changes in gene expression are represented as gradations according to their FDR values, which were determined using the rank product method [34,35]. Overall, both genome reduction and heat shock exerted broad effects on transcriptional reorganization: the expression levels of a number of genes were changed (Figure 3). A small number of regulators showed remarkable, independent transcriptional changes; for example, *gadX* was induced only by genome reduction (A) and *rpoD* only by heat shock (B). It indicated that genome structural changes might activate the primary activator of the *gad* system but fairly influenced the global transcriptional factor (sigma factor). In addition, a comparable number of regulators were disturbed by both perturbations in a correlated manner: those induced by genome reduction were also induced by heat shock. The changes in these overlapping perturbed regulators occurred in either the same or the reverse direction; *e*.*g*., the expression level of *phoB* was upregulated by both genome reduction and heat shock (orange in both A and B), whereas, the expression level of *gadE* was upregulated by genome reduction but repressed by heat shock (orange in A, purple in B).

To identify these regulators (regulatory networks) upstream of genes with marked changes in transcription induced by either genome reduction or heat shock, a gene set enrichment analysis (GSEA) was applied [36]. Among the 175 regulatory networks controlled by 5 sigma factors and 170 TFs, 24 and 12 networks showed clear transcriptional changes (FDR < 0.25) associated with genome reduction and heat shock, respectively (Table 1; details in Additional file 2: Table S1). Heat shock interrupted approximately equal numbers of the induced and repressed regulatory networks (7 *vs*. 5), whereas genome reduction triggered many more upregulated than downregulated networks (22 *vs*. 2). Moreover, most of the regulatory networks that responded to heat shock were highly sensitive to genome reduction as well (9 out of 12). These overlapping regulatory networks (*e*.*g*., *rpoH* and *purR*) showed a marked tendency to undergo transcriptional changes in the same direction (*i*.*e*., increased or decreased) in response to genomic and heat shock perturbations. The exception (with changes in opposite directions) was found in the network controlled by *gadX*, where the downstream genes were induced by genome reduction but suppressed by heat shock (FDR < 0.02).

**Table 1:** Regulatory networks with significant perturbations

**Perturbation**	**Direction**	**Locus tag**	**Name of TF**	**Size**	**FDR q-val**
Genome reduction	Increased	b1951	rcsA	26	0.0000
		b2217	rcsB	29	0.0000
		b3938	metJ	15	0.0000
		b3516	gadX	25	0.0000
		b3512	gadE	49	0.0000
		b0889	lrp	83	0.0038
		b3461	rpoH	150	0.0065
		b0761	modE	45	0.0061
		b1237	hns	136	0.0145
		b3261	fis	216	0.0513
		b2193	narP	49	0.0870
		b1221	narL	113	0.1043
		b3912	cpxR	51	0.0972
		b2531	iscR	26	0.1580
		b0399	phoB	36	0.1572
		b1712	ihfA	191	0.1477
		b2741	rpoS	215	0.1448
		b0912	ihfB	191	0.1398
		b0076	leuO	20	0.1703
		b1130	phoP	48	0.1627
		b1334	fnr	272	0.1687
		b1531	marA	36	0.2127
	Decreased	b1658	purR	31	0.0071
		b0683	fur	81	0.0201
Heat shock	Increased	b3461	rpoH	150	0.0000
		b3912	cpxR	51	0.0004
		b3938	metJ	15	0.0026
		b2193	narP	49	0.0837
		b0399	phoB	36	0.1384
		b0761	modE	45	0.2134
		b0076	leuO	20	0.2345
	Decreased	b1658	purR	31	0.0000
		b1275	cysB	19	0.0000
		b0683	fur	81	0.0000
		b3516	gadX	25	0.0123
		b3868	glnG	44	0.0118

The information pertaining to transcription factors (TFs), sigma factors and the downstream genes under their control is from RegulonDB. The transcriptional changes in the downstream genes were evaluated with gene set enrichment analysis (GSEA) [36], and the disturbed regulatory networks were determined accordingly (FDR <  0.25). Perturbation and Direction refer to the type of disturbance to the transcriptome and the directionality of the changes in gene expression, respectively. Locus tag, Name of TF, Size and FDR q-val represent the gene ID, the corresponding regulator gene, the number of regulated downstream genes and the statistical significance, respectively.

Taken together, the transcriptional reorganization observed in the regulatory networks was not only secondary to the expression changes in the regulators (*e*.*g*., *rpoH*) but also occurred in networks controlled by the regulators of steady expression (*e*.*g*., *purR*). The latter case suggested either weakened transcriptional control by the regulators or a high level of sensitivity in the downstream genes (*i*.*e*., slight transcriptional changes in the regulators resulted in profound transcriptional changes in the downstream genes). The discovery of regulatory networks (and/or regulators) that were universally involved in both perturbations indicated that transcriptional reorganization responds similarly to both endogenous and exogenous disruptions.

### Genome location dependency and gene category enrichment of highly responsive genes

**Figure 4:**
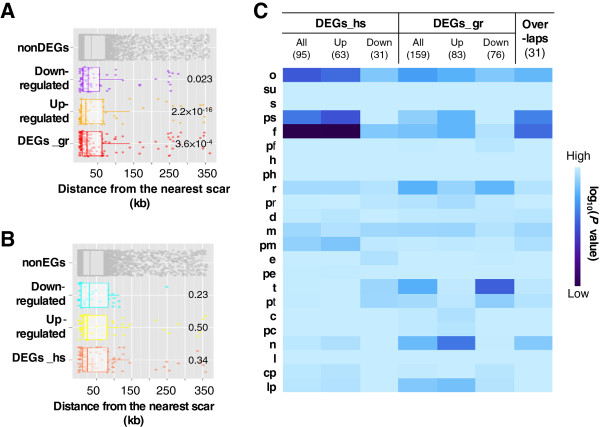
**Differentially expressed genes (DEGs). A-B**. Distance between genes and genomic scars. The distance from each gene to the nearest genomic scar resulting from genome reduction is plotted. The genes with conserved (nonDEGs), increased, or reduced expression caused by genome reduction are shown in gray, orange, and purple, respectively (**A**). The genes with conserved (nonDEGs), upregulated, or downregulated expression due to heat shock are shown in gray, yellow and cyan, respectively (**B**). All the genes of the DEGs_gr and DEGs_hs are shown in red and brown, respectively. Significance was determined with the Wilcoxon-Mann–Whitney test, and the *P*-values are indicated. **C**. Enriched gene categories. The DEGs were divided into 23 gene categories, according to a previous report [37]. One-tailed binominal tests were performed to determine the significance of the enrichment of each category. The log-scale *P*-values are displayed as a heat map with gradations of blue; darker blue indicates higher significance.

Genes with significant transcriptional changes (differentially expressed genes, DEGs) among the 3710 common genes were also determined with the rank product method. In all, 159 and 95 genes were identified that showed either up- or downregulated expression in response to genome reduction (DEGs_gr) or heat shock (DEGs_hs), respectively (FDR < 0.001). The gene names, gene categories, locus tags, and directions of the transcriptional changes are summarized in Additional file 3: Table S2, and the genome locations are marked in Additional file 1: Figure S1. An analysis of the distances between the DEGs and the genome scars caused by genome reduction showed that the DEGs_gr, but not the DEGs_hs, were located much closer to the nearest genome scar than were the genes with conserved expression (nonDEGs) (*P*  <  0.001) (Figure 4A and B). This tendency indicated that the genomic disturbance caused by genome reduction brought about considerable transcriptional changes in neighboring genes. In particular, the transcriptional changes were highly significant in the direction of upregulation (Figure 4A, orange), which suggests that the removal of the dispensable genetic segment might increase the expression efficiency of the neighboring genes. We assumed that the deleted genes most likely perturbed the local superhelical density and thereby affected the transcriptional activity of the neighboring genes, although the functions of the deleted genes near the deletion junctions or mutations remain to be further examined.

The gene categories that were enriched in the DEGs were analyzed to address the question of whether the genomic and heat shock perturbations had affected any specific gene categories (details in Additional file 3: Table S2 and Additional file 4: Table S3). The heat map (Figure 4C) illustrates that the heat shock affected only a limited number of gene categories (*i*.*e*., Unknown function and Factor) with high significance (*P* < 0.001). However, the genome reduction influenced a relatively large number of gene categories with diverse directions in transcriptional changes; for example, the transporter genes (t) and the RNA-coding genes (n) were highly enriched in the DEGs_gr in the directions of down- (*P* < 0.001) and upregulation (*P* < 0.002), respectively. Only the “Unknown function” (o) category was significantly enriched in both the DEGs_gr and the DEGs_hs (All, *P* < 0.05). This result suggested that the genes with unknown functions were highly sensitive to both genomic and environmental perturbations, as might result from a loose control of gene expression. The genes in the Factor category (f), of which most are heat shock proteins (chaperones), were not only enriched in the upregulated DEGs_hs (Up, *P* < 0.001), as extensively reported, but were also detected in the upregulated DEGs_gr (Up, *P* < 0.05). As a result, Factor (f) was the most highly enriched category among the 31 overlapping genes (*P* < 0.001). Intriguingly, some well-known molecular chaperones, such as *clpB* and *ibpA*, were highly responsive to both perturbations (Additional file 3: Table S2), indicating a universal response mechanism to internal and external stressors in these genes.

### Positive correlations in changes of gene expression

**Figure 5:**
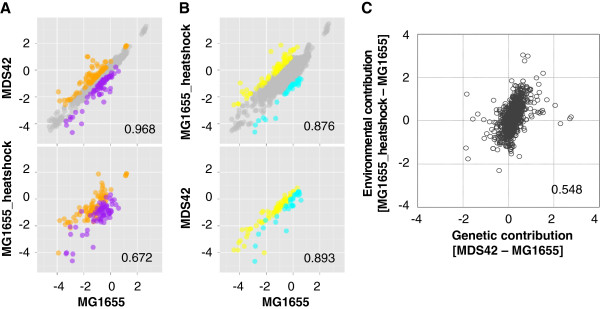
**Positive correlations between the genomic and environmental contributions.** Firstly, scatter plot of the expression levels of 3710 common genes in MDS42 and MG1655 is shown (**A**, top panel). A total of 159 genes with differential expression in MDS42 caused by genome reduction (DEGs_gr) are highlighted, including 83 with increased expression (in orange) and 76 with reduced expression (in purple), respectively. The expression levels of these 159 genes (DEGs_gr) in MG1655 under the regular and heat shock conditions (MG1655 *vs*. MG1655_heatshock) are additionally compared (**A**, bottom panel). Secondly, scatter plot of the expression levels of 3710 common genes in MG1655 in the presence or absence of heat shock treatment is shown (**B**, top panel). A total of 95 genes with differential expression due to heat shock (DEGs_hs) in MG1655 are highlighted, including 64 upregulated (in yellow) and 31 downregulated (in cyan) genes, respectively. The expression levels of these 95 genes (DEGs_hs) in both MG1655 and MDS42 under regular conditions (MG1655 *vs*. MDS42) are also compared (**B**, bottom panel). Finally, positive correlations between the genetic and environmental contributions to the transcriptional changes of total 3710 common genes are shown (**C**). The genetic contribution refers to the changes in gene expression caused by genome reduction, *i*.*e*., the subtraction of the expression level in MG1655 from the expression level in MDS42. The environmental contribution refers to the changes in gene expression due to heat shock in MG1655, *i*.*e*., the subtraction of the expression level in MG1655 from the expression level in MG1655_heatshock. The expression level is determined as log_10_ (mRNA concentration), with the unit of pM. The Pearson’s correlation coefficients are indicated.

The directions of the transcriptional changes demonstrated by the DEGs were further analyzed, in particular, on the gene expression patterns by means of correlations. In a global view, the DEGs_gr (Figure 5A, upper) also showed remarkable transcriptional changes in response to heat shock (Figure 5B, upper), as evidenced by the significantly lower correlation of the DEGs_gr (0.672) compared with that (0.876) of the 3710 common genes (*P* < 0.001, Additional file 1: Figure S2). Similarly, the DEGs_hs showed large transcriptional changes in response to genomic perturbation (Figure 5B, bottom); their correlation (0.893) was significantly lower than that (0.968) of the 3710 common genes (*P* < 0.001, Additional file 1: Figure S2). Furthermore, the upregulated DEGs_hs (Figure 5A, yellow) were also induced by genome reduction (Figure 5B, yellow), and the upregulated DEGs_gr (Figure 5A, orange) were also induced by heat shock (Figure 5B, orange). These correlated patterns of changes in gene expression deduced from the overall evaluation of the DEGs (Figure 5A and B) implied that the overlapping genes (Figure 4C) might play a crucial role in producing such transcriptional fluctuation because not all the DEGs changed significantly when moving to the other condition. Among the 31 overlapping genes (Figure 4C, Additional file 4: Table S3), 20 and 10 genes with up- or downregulated expression in response to genome reduction were up- or downregulated by heat shock in the same direction, respectively. Only a single gene (*yafF*, a conserved protein) reversed the direction of its transcription; *i*.*e*., it was induced by heat shock but repressed by genome reduction. The overall directional similarities implied that the genes that were sensitive to genomic disturbance tended to be highly responsive to external perturbation and *vice versa*.

Furthermore, the transcriptional changes of the 3710 genes that were induced by genome reduction were plotted against those that were induced by heat shock (Figure 5C). A clear, positive correlation between the transcriptional changes produced by the genetic and environmental contributions was observed (*P* < 0.001, Additional file 1: Figure S2). In other words, larger degrees of transcriptional fluctuation triggered by genetic interruption were correlated with greater changes in gene expression induced by environmental perturbation. The positive correlation between these two contributions to the transcriptome implies somehow a linkage in transcriptional reorganization in response to both genetic and environmental stresses.

### Antagonistic epistasis in the transcriptome

**Figure 6:**
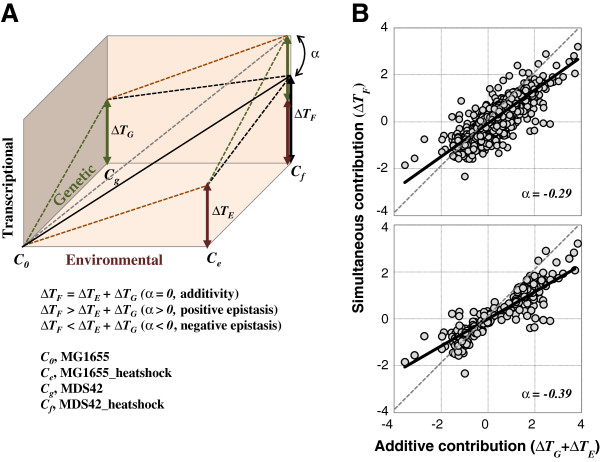
**Epistasis between genomic and environmental perturbations. A**. Schematic illustration of epistasis and additivity. The environmentally and genetically induced transcriptional changes are defined as *ΔT*_*E*_ and *ΔT*_*G*_, respectively. When genetic and environmental perturbations occurred together, the simultaneous change in expression is defined as *ΔT*_*F*_. The relationships among these three parameters were described with the indicated equations, where *α* represents the magnitude of the epistatic effect. **B**. Antagonistic epistasis in the transcriptome. The sum of the transcriptional changes caused separately by genetic and environmental perturbations (the additive contribution, *ΔT*_*E*_ + *ΔT*_*G*_) is plotted against the transcriptional changes caused simultaneously by both perturbations (the simultaneous contribution, *ΔT*_*F*_), *i*.*e*., the changes in gene expression between MDS42_heatshock and MG1655. The 3710 common genes and the genes showing significant fluctuations induced by either genetic or environmental perturbation (both DEGs_gr and DEGs_hs) are plotted in the upper and bottom panels, respectively. The values of *α* were calculated as follows: *α* = 1 – *k*, where *k* is the slope of the dot plot fit by linear regression, as indicated by the solid black line.

Theoretically, the positive correlation in the transcriptional changes triggered by the genetic and environmental interruptions (Figure 5C) potentially had a tendency to amplify the magnitude of the changes in gene expression that occurred in response to both genetic and environmental perturbations. For instance, the gene expression levels of *dnaJ* were approximately 2.6 and 30 folds up-regulated due to genome reduction and heat shock, respectively. Owing to the positive correlation, the change in the expression of *dnaJ* was assumed to be multiplied, up to 78 folds, by the two simultaneously occurred perturbations. Actually, it was a 21-fold increase, a highly suppressed value, in the transcriptional change. To understand such repressed change in gene expression, the genetic concepts of epistasis and additivity were employed, as represented schematically in Figure 6A. These concepts are widely used to determine whether the final fitness or output is simply the sum of the effects of two genes (additivity) or whether one gene partially inhibits (negative epistasis) or enhances (positive epistasis) the effects of the other gene. The extended definition of epistasis could be applied to the interactions among the chemicals and molecules in highly complex biochemical reactions [38]. Here, the two analyzed effects were the transcriptional changes contributed by genome reduction (∆*T*_*G*_) and heat shock (∆*T*_*E*_) individually, and the final output was defined as the transcriptional changes that were produced when the genomic and heat shock perturbations occurred simultaneously (∆*T*_*F*_), *i*.*e*., the reduced genome undergoing heat shock (MDS42_heatshock). The slopes represent the transcriptional changes (∆*T*_*F*_) perturbed by the genome and environment in an additive or epistatic manner (Figure 6A, indicated by the solid black line and the broken gray line). The difference in the slopes, defined as *α*, represented the relative magnitude of the difference (repressed or increased) between the additive and simultaneous contributions.

Accordingly, the relationship between the contributions of genome reduction and heat shock to transcriptome was subsequently analyzed in a genomic view. Considering the difference in the number of genes in MG1655 and MDS42, the common genes (3710 genes) shared with both strains were applied in the following analyses. The transcriptional changes due to these two independent contributors (genome and environment, as shown in Figure 5C) were added, and the sum was considered to be the additive contribution (∆*T*_*G*_ + ∆*T*_*E*_). The changes in gene expression between MDS42_heatshock and MG1655 represented simultaneous genetic and environmental interruptions (simultaneous contribution, ∆*T*_*F*_). The additive contribution was then plotted against the simultaneous contribution, as shown in Figure 6B. The simultaneous contribution showed a smaller magnitude of transcriptional changes than did the additive contribution (*α* < 0) (Figure 6B, upper panel), approximately 30% suppressive effect (*α* = −0.29, *p* < 0.001). It strongly indicated a negatively compensated relationship between the genomic and environmental contributions to the changes in the transcriptome, as so-called “antagonistic epistasis”. Moreover, the magnitude of the compensatory effect became larger (*α* = −0.39) in the gene groups of the DEGs_gr and the DEGs_hs (Figure 6B, bottom panel), suggesting that the negative epistasis became more significant, approximately 40%. Both the genetic and environmental perturbations alone triggered fluctuations in the transcriptome, whereas either perturbation repressed or limited the fluctuation caused by the other when both occurred simultaneously. These results indicated that negative epistasis may be universally involved in biological processes, although the underlying mechanisms and theoretical principles remain to be determined.

## Discussion

This study demonstrated a considerable interaction between the genetic and environmental contributions to the changes in the transcriptome, as evidenced by the identical preferred chromosomal periodicity, the directional similarities in the transcriptional changes, the overlaps between the perturbed regulatory networks, the positive correlation in transcriptional changes induced by the genetic and environmental contributions and the negative epistasis. Thus, our multilevel comparative analysis provided a global comparison of the effects of genomic and heat shock perturbations to the *E*. *coli* transcriptome. A more comprehensive evaluation based on the additional experiments under cross multiple conditions and a cross validation using the fruitful reported data sets are essential to figure out the common features of the genomic and environmental contributions to the reorganization in transcriptome.

The transcriptome analysis caught the genome reduction effect. The deleted regions were assumed to have crucial functions, on the basis of our novel observations of the reduced genome, along with the previous reports of its advantages in various applications [17,21,23] but its limited evolvability [20]. As these regions were largely occupied by genes that were derived from the IS/phage, that encoded structural components, or that had unknown or predicted functions, it highlighted the potential importance of these genes. We observed that the deletions altered the transcriptional efficiency, the stability of transcriptional patterns and the specificity of transcriptional changes. The high average levels of gene expression (Figure 1B) and the strong significance of the DEGs_gr upregulation (Figure 4A) indicated that the elimination of the redundant genome sequence may save materials and energy, leading to increases in the transcriptional efficiency of the remaining genes. The observation that the fixed chromosomal periodicity (Figure 2C and D) was identical to the number of chromosomal macrodomains [33] indicated that the global transcriptional pattern may be stabilized by the complete removal of the flexible IS/phage regions. The slight but significant increase in the cell growth rate most likely resulted from this steady expression pattern, which potentially accelerated cell division [39]. The significant enrichment of genes of unknown function in the DEGs_gr and DEGs_hs (Figure 4C, Additional file 4: Table S3) suggested that these genes are easily disturbed. Additional analyses revealed an apparent bias in the genomic positions of the unknown function genes; these genes were preferentially located in areas near the deleted regions (Additional file 1: Figure S3), which explained the finding of genomic scar dependency in the DEGs_gr (Figure 4A). The genomic distribution of the functionally undefined genes reflected shared gene organization strategies [40] that were most likely shaped by evolution. Taken together, the data suggest the possibility of a relationship between the identities of the deleted genes (in particular, the genes of unknown function), the chromosomal distribution of the deleted genes, the global expression patterns (periodicity), and the cell growth (fitness).

The genomic and environmental contributions to transcriptome reorganization indicated a negative epistatic relationship (Figure 6B), that is, a negatively compensative relation of the transcriptional changes caused by the two contributors. Such relationship would inhibit the transcriptional fluctuation that was amplified by the positive correlation between the changes in gene expression triggered by the genetic and environmental interruptions (Figure 5C). Epistasis has largely been studied within the framework of genetics, *i*.*e*., how a mutation in one gene masks the phenotypic effect of a mutation in another gene [41,42]. The present study was the first to compare the contributions of the genome and the environment to the transcriptome and to include an analysis of the antagonistic (negative) epistasis involved in the reorganization of transcriptome. Because MDS42 was synthetically constructed and is therefore not a product of evolution in the wild nature, the antagonistic epistatic relationship between the genomic and environmental contributions suggested a universal innate principle of cell biology. This principle could be described as the need to restrict the consequences of two positively correlated effects to physiologically tolerable limits. The magnitude of the compensatory effect detected in the DEGs was larger than that in the 3710 common genes (Figure 6B) strongly supported this theory. The adage that the sum is less than its parts (*e*.*g*., ∆*T*_*G*_ + ∆*T*_*E*_ < ∆*T*_*F*_) appears to be applicable to the ability of living organisms to survive in severe environments for millions of years and to an earlier report of the robustness and/or plasticity of living systems [43], although more data are required to support this hypothesis.

## Conclusion

This study investigated the relationship between the genetic and environmental contributions to the bacterial transcriptome. Intriguingly, in addition to similarities and overlaps in periodicity, regulatory networks, DEGs and directionality, both positive correlations and negative epistasis between the genomic and environmental contributions to the global transcriptional activity were observed. These results linked the genome and the environment at the level of the transcriptome and revealed the epistatic nature of the genome-wide transcriptional reorganization that occurs universally in living cells in response to both endogenous and exogenous disturbances.

## Materials and methods

### Strains and culture conditions

The wild-type *E*. *coli* strain MG1655 was provided by the National BioResource Project, National Institute of Genetics, Shizuoka, Japan. The reduced-genome *E*. *coli* strain MDS42 was purchased from Scarab Genomics. The genome sequences of MG1655 and MDS42 were retrieved from the information deposited in the GenBank and DDBJ databanks and have accession IDs of U00096 and AP012306, respectively. When comparing the two deposited genome sequences, we found that 696 genes presented in MG1655 were absent in MDS42 (*i*.*e*., 696 deleted genes), consistent with the original report [17]. In addition, 22 genes showed mismatched sequences between two genomes (*i*.*e*., 22 mutated genes). Thus, a total of 3710 genes (if including *oriC*, then 3711 genes) of perfect matched sequences were determined to be common between the two strains. The bacterial cells were cultured in 5 mL of M63 minimal medium (62 mM K_2_HPO_4_, 39 mM KH_2_PO_4_, 15 mM (NH_4_)_2_SO_4_, 2 μM FeSO_4_·7H_2_O, 15 μM thiamine hydrochloride, 203 μM MgSO_4_·7H_2_O and 22 mM glucose) at 37°C with shaking at 200 rpm in a BR-21FP air incubator (Taitec).

### Cell culture conditions for the precise measurement of mRNA levels and growth rates

The cell cultures were established from a glycerol stock with two preculture steps, and serial transfers were performed during the exponential phase. The dilution rate needed to keep the final cell concentration at approximately 1-2 × 10^8^ cells/mL was calculated according to the growth rate observed during the previous day. The initial cell concentration (*C0*) was determined on the basis of the concentration of the preculture used for transfer and the dilution rate. The final cell concentration (*Ct*) was measured using a flow cytometric analysis (Canto II; Becton, Dickinson and Company) of the remainder of the cell cultures that had been sampled for microarray analysis. The growth rate was calculated according to the following formula: ln(*Ct*/*C0*)/*t*, where *Ct*, *C0*, and *t* represent the final and initial cell concentrations (cells/mL) and the culture time (h), respectively. This formula has been previously applied in the quantitative evaluation on bacterial growth in exponential phase [28,29,44].

### Heat shock experiment

As previous studies showed that heat shock response in *E*. *coli* was evidently induced within a few minutes (*i*.*e*., 5–10 minutes) after the temperature upshift from 30–37°C to 42–50°C [9,45-47], the condition for heat shock experiment was determined as a 5-min incubation following the temperature upshift from 37°C to 45°C [28]. Exponentially growing cells at approximately 10^8^ cells/mL were rapidly transferred to an adjacent water bath incubator (Personal-11EX; Taitec) set at 45°C. Following a 5-min incubation at 45°C, the cell culture was immediately poured into a cold phenol-ethanol solution to prepare the samples for microarray analysis. Each heat shock experiment was performed separately to enable the precise control of the timing of the heat shock to ensure that the mRNA levels accurately reflected the stress response.

### Microarrays and data extraction

A high-density DNA microarray covering the entire genome of the *E*. *coli* W3110 strain was utilized [26,27] as described elsewhere [28,29]. The sample preparation, the microarray analysis with the Affymetrix GeneChip system, and the data extraction based on the finite hybridization (FH) model [26] were performed as previously described [29]. To avoid any significant errors resulting from the diverse signal intensities of the GeneChips (*i*.*e*., to minimize experimental errors), only array results with highly similar distributions of probe fluorescence intensities were included in the subsequent expression analysis. The results of 20 arrays for the two strains under the two conditions were used (7 and 3 replicas of exponential growth under 37°C and 45°C heat shock conditions, respectively). The raw data from these 20 microarrays were deposited in the NCBI Gene Expression Omnibus database under the GEO Series accession number GSE33212 (http://www.ncbi.nlm.nih.gov/geo/query/acc.cgi?acc=GSE33212). The gene names are based on the genome information for W3110 and represent the genes in common between strains W3110 and MG1655 [37]. The entire dataset of gene names and categories is from GenoBase, Japan (http://ecoli.aist-nara.ac.jp/gb6/Download.html).

### Computational analyses and graphics

The transcription levels were determined as the log-scale mRNA concentrations (pM). The transcriptional changes (fluctuations) were calculated as the difference between the two transcription levels. Although several methods for the determination of chromosomal periodicity have been reported [4,32], a simple approach was used here. Each expression value that had been determined with the method described in the previous section was projected onto the genome site that corresponded to the gene position, using 100-base bins. Next, the series of expression levels along the genome was smoothed with a moving average of 500 bins (50 kb). The periodicity was calculated using a standard Fourier transform, and the significance of the periodicity was assessed with the chi-squared test. The approximate line of the periodicity was calculated using the highest peak (statistic significance) of the periodogram and was fitted by minimizing the square error between the approximate line and the series of expression values. Gene set enrichment analysis (GSEA) was performed according to the original report [36] using the available online tools (http://www.broadinstitute.org/gsea/index.jsp). The TF and sigma factor gene regulation datasets were from RegulonDB. To compare the responses of MG1655 and MDS42, we limited the gene sets to those genes that were included among the 3710 common genes. The pre-ranked gene lists used as the input for GSEA comprised genes filtered by the absolute value of their expression difference between MG1655_hs and MG1655 (or MDS42 and MG1655). The regulatory networks were visualized using Gephi software (http://gephi.org). Subsequently, the Bioconductor software package RankProd [34], which is based on the rank product method, was employed to identify the differential gene expression caused by genome reduction and the heat shock response. The rank product method is a nonparametric statistical method derived from biological reasoning that detects items that are consistently ranked high or low in a number of lists. An advantage of this method is its ability to identify biologically relevant expression changes among a relatively small number of samples [35]. Finally, the statistical analyses, with the exception of GSEA, were performed using R software [48] (http://www.r-project.org), and the array plot (heat map) of the gene categories was constructed using Mathematica 8 (Wolfram Research).

## Abbreviations

*E*. *coli*: *Escherichia coli*; TF: Transcription factor; bp: Base pair; kb: Kilobase; gr: Genome reduction; hs: Heat shock; DEGs: Differentially expressed genes; FDR: False discovery rate; GSEA: Gene set enrichment analysis.

## Competing interests

The authors declare that there is no competing interest.

## Authors’ contributions

BWY and TY conceived the research; BWY designed and performed the experiments; BWY, SS and TY analyzed the data; FK, HM and TY provided the analytic tools; and BWY, SS and TY wrote the manuscript. All the authors read and approved the final manuscript for publication.

## Supplementary Material

Additional file 1 **Figure S1.** Chromosomal locations of the DEGs. **Figure S2**. Statistical analysis. **Figure S3**. Genes of unknown function. **Figure S4**. A close-up view of the transition in major peaks. Click here for file

Additional file 2 **Table S1.** Detailed results of the gene set enrichment analysis. Click here for file

Additional file 3 **Table S2.** List of the DEGs. Click here for file

Additional file 4 **Table S3.** Analysis of the enriched gene categories. Click here for file
