# Supplementary material for: Multilevel comparative analysis of the contributions of genome reduction and heat shock to the Escherichia coli transcriptome
Source: BMC Genomics. 2013 Jan 16;14:25. doi: 10.1186/1471-2164-14-25 (PMC3553035; doi:10.1186/1471-2164-14-25)
Supplement: Additional file 1 — Figure S1. Chromosomal locations of the DEGs. Figure S2. Statistical analysis. Figure S3. Genes of unknown function. Figure S4. A close-up view of the transition in major peaks. [file 1471-2164-14-25-S1.pdf]

Multilevel comparative analysis of the contributions of genome reduction and heat shock to the *Escherichia coli* transcriptome

Ying and Seno et al

Supplementary figures and figure legends

Supplementary figure legends

Figure S1 Chromosomal locations of the DEGs. The positions of the DEGs_gr and DEGs_hs on the genome are indicated. The black line indicates the MG1655 genome, and the deleted segments are indicated by gray boxes. The up- and downregulated DEGs_gr are indicated in orange and purple, respectively (**A**). The up- and downregulated DEGs_hs are indicated in yellow and cyan, respectively (**B**). The right- and left-pointing arrowheads represent the genes encoded on the forward and complementary strands, respectively.

Figure S2 Statistical analysis. A. Significance test of the correlation of the DEGs_gr. To test for significance, 159 genes were randomly selected, and their correlation coefficients were calculated. The histogram shows the results of 10,000 permutations of random sampling, and the red points indicate the correlation coefficients of 159 DEGs_gr between MG1655 and MG1655_heatshock. **B**. Significance test of the correlation of the DEGs_hs. To test for significance, 95 genes were randomly selected, and their correlation coefficients were calculated. The histogram shows the results of 10,000 permutations of random sampling, and the red points indicate the correlation coefficients of the gene expression level of the 95 DEGs_hs between MG1655 and MDS42. The *P*-values are indicated. **C**. Significance test of the overall correlation of the gene expression levels between MG1655 and MDS42. To test for significance, a dummy vector of expression values was randomly generated while maintaining a certain level of correlation in the gene expression values with MG1655. This figure demonstrates that the effects of genetic interruption and external perturbation are not independent. **D**. The significance test of the overall correlation between MG1655 and MG1655_heatshock. A dummy vector was randomly generated as above and evaluated. The *P* values are indicated.

Figure S3 Genes of unknown function. All 3710 genes were divided into two categories, “Unknown” and “not Unknown,” according to Reliy *et al.* [45]. The distances from each gene to the genomic scars are plotted. The “Not Unknown” and “Unknown” categories are shown in the upper panel. The “Unknown” genes were classified as DEGs or nonDEGs and are plotted in the bottom panel. Statistical significance was calculated with the Wilcoxon-Mann-Whitney test, and the *P*-values are indicated.

Figure S4 A close-up view of the transition in major peaks. The vertical, broken lines in red indicate the major peaks at 663 and 773 kb, respectively.

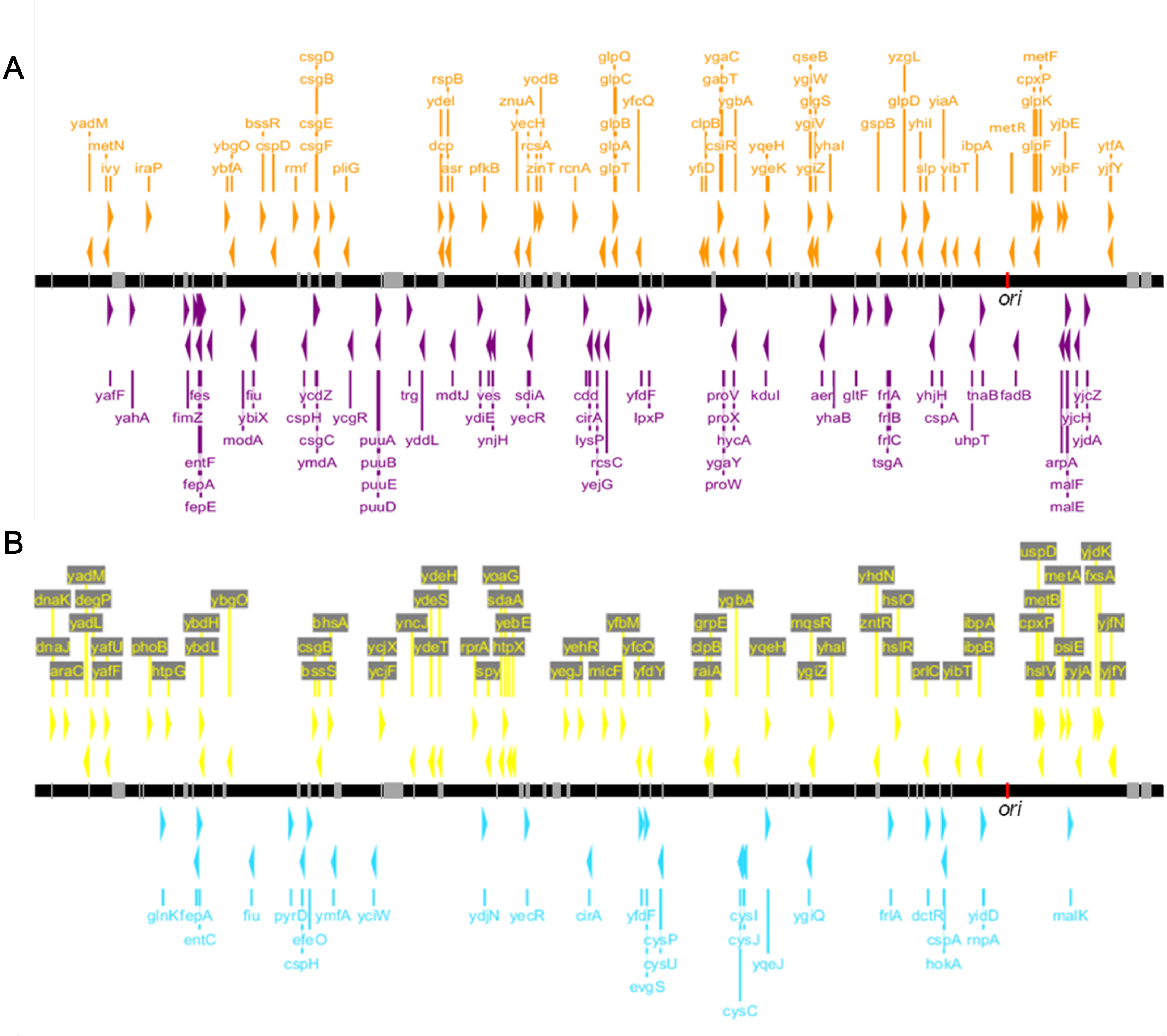
A
csgD
csgB
csgE
csgF
yadM
metN
ivy
iraP
bssR
ybgO
cspD
ybfA
rmf
pliG
rspB
ydeI
dcp
asr
pfkB
yodB
znuA
yecH
rcsA
zinT
rcnA
glpQ
glpC
glpB
glpA
glpT
yfcQ
ygaC
gabT
ygbA
clpB
csiR
yfiD
yqeH
ygeK
qseB
ygiW
glgS
ygiV
yhaI
ygiZ
yzgL
glpD
yiaA
gspB
yhiI
slp
yibT
ibpA
metF
cpxP
glpK
metR
glpF
yjbE
yjbF
ytfA
yjfY
ori
yafF
yahA
fes
fimZ
entF
fepA
fepE
fiu
ybiX
modA
ycdZ
cspH
ycgR
csgC
ymdA
puuA
puuB
puuE
puuD
trg
yddL
mdtJ
ves
ydiE
ynjH
sdiA
yecR
cdd
cirA
lysP
rcsC
yejG
yfdF
lpxP
proV
proX
hycA
ygaY
proW
kduI
aer
yhaB
gltF
frlA
frlB
frlC
tsgA
yhjH
cspA
tnaB
uhpT
fadB
yjcZ
yjcH
yjdA
arpA
malF
malE
B
yadM
dnaK
degP
yadL
dnaJ
yafU
araC
yafF
phoB
htpG
ybdH
ybdL
ybgO
bhsA
csgB
bssS
ycjX
ycjF
ydeH
ydeS
yncJ
ydeT
yoaG
sdaA
yebE
rprA
htpX
spy
yehR
yegJ
yfbM
micF
yfcQ
yfdY
ygbA
grpE
clpB
raiA
yqeH
mqsR
yhaI
ygiZ
yhdN
hslO
zntR
hslR
ibpA
ibpB
prlC
yibT
uspD
yjdK
metA
fxsA
metB
cpxP
psiE
yjfN
hslV
ryjA
yjfY
ori
glnK
fepA
entC
fiu
pyrD
ymfA
efeO
cspH
yciW
ydjN
yecR
cirA
yfdF
cysP
cysU
evgS
cysI
cysJ
cysC
ygiQ
yqeJ
frlA
dctR
cspA
hokA
yidD
rnpA
malK

Figure S1

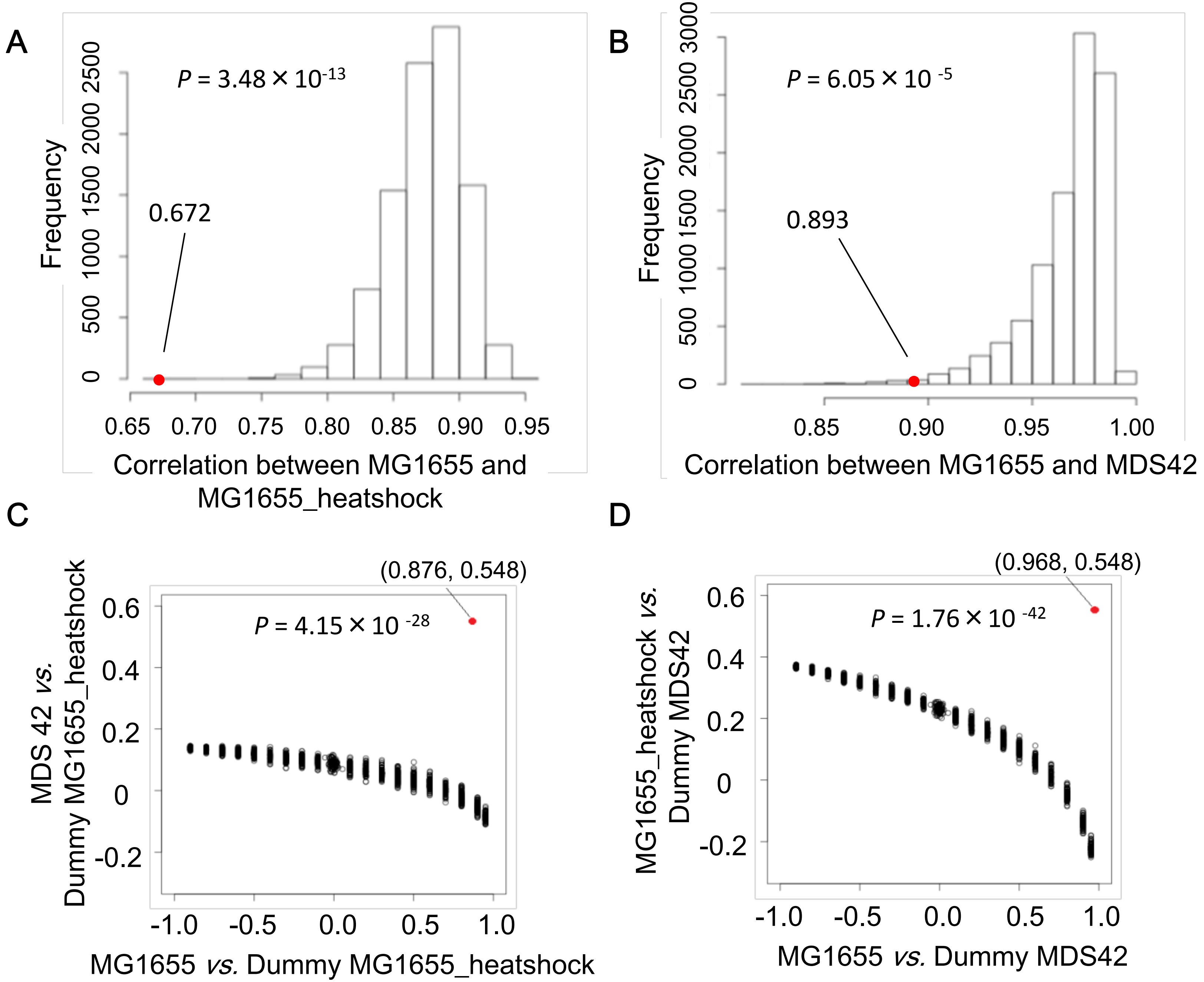
A
P = 3.48 × 10-13
0.672
Frequency
0 500 1000 1500 2000 2500
0.65 0.70 0.75 0.80 0.85 0.90 0.95
Correlation between MG1655 and MG1655_heatshock
B
P = 6.05 × 10 -5
0.893
Frequency
0 500 1000 1500 2000 2500 3000
0.85 0.90 0.95 1.00
Correlation between MG1655 and MDS42
C
(0.876, 0.548)
P = 4.15 × 10 -28
MDS 42 vs. Dummy MG1655_heatshock
-0.2 0 0.2 0.4 0.6
-1.0 -0.5 0.0 0.5 1.0
MG1655 vs. Dummy MG1655_heatshock
D
(0.968, 0.548)
P = 1.76 × 10 -42
MG1655_heatshock vs. Dummy MDS42
-0.2 0 0.2 0.4 0.6
-1.0 -0.5 0.0 0.5 1.0
MG1655 vs. Dummy MDS42

Figure S2

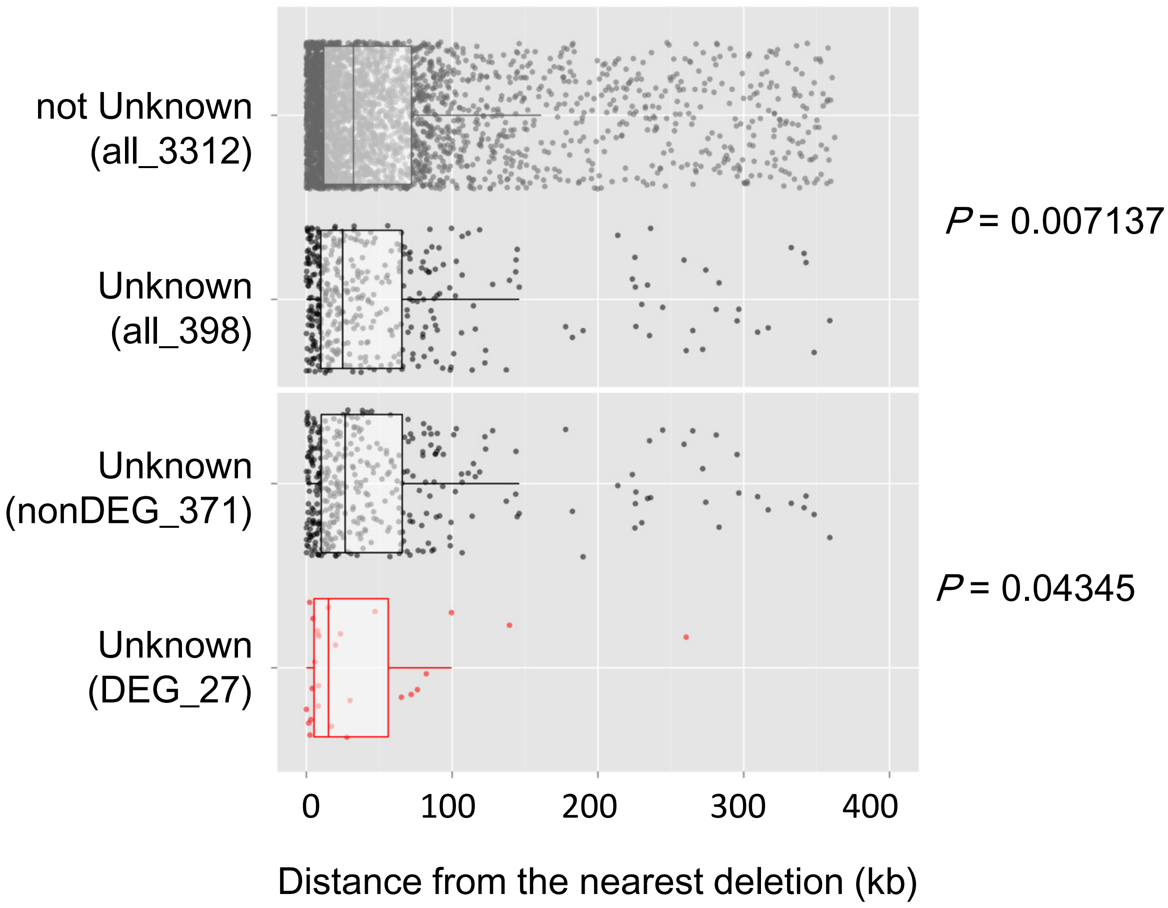
not Unknown
(all_3312)
Unknown
(all_398)
Unknown
(nonDEG_371)
Unknown
(DEG_27)
P = 0.007137
P = 0.04345
0
100
200
300
400
Distance from the nearest deletion (kb)

Figure S3

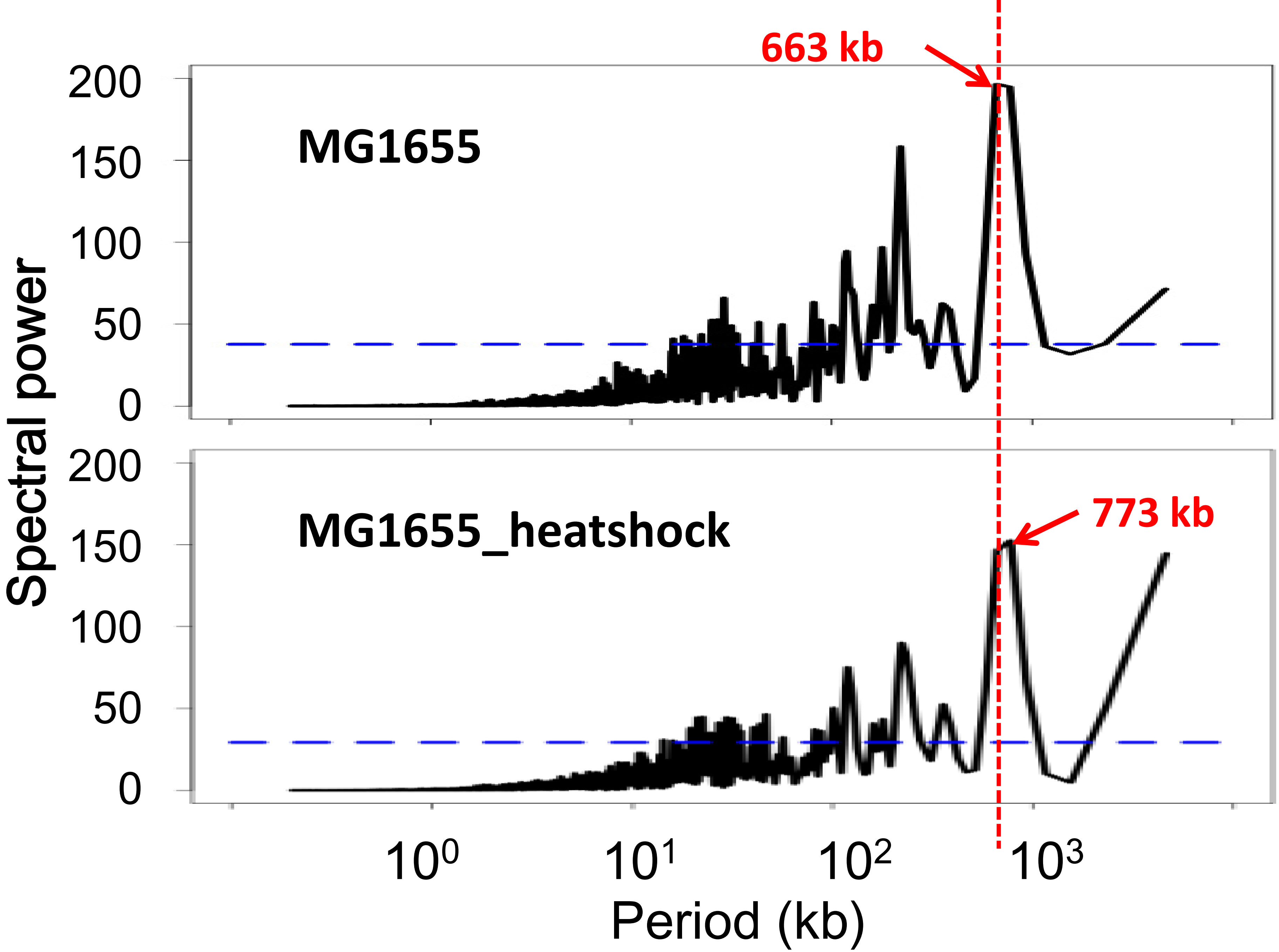
663 kb
MG1655
773 kb
MG1655_heatshock
Spectral power
200
150
100
50
0
10^0
10^1
10^2
10^3
Period (kb)

Figure S4
